# Persistent Interactions with Bacterial Symbionts Direct Mature-Host Cell Morphology and Gene Expression in the Squid-Vibrio Symbiosis

**DOI:** 10.1128/mSystems.00165-18

**Published:** 2018-10-02

**Authors:** Natacha Kremer, Eric J. Koch, Adil El Filali, Lawrence Zhou, Elizabeth A. C. Heath-Heckman, Edward G. Ruby, Margaret J. McFall-Ngai

**Affiliations:** a Department of Medical Microbiology and Immunology, University of Wisconsin—Madison, Madison, Wisconsin, USA; b Laboratoire de Biométrie et Biologie Evolutive, Université de Lyon, Université Lyon 1, CNRS, UMR 5558, Villeurbanne, France; c Kewalo Marine Laboratory, University of Hawaii—Manoa, Honolulu, Hawaii, USA; Australian Institute of Marine Science

**Keywords:** *Euprymna scolopes*, *Vibrio fischeri*, accommodation, development, persistent symbiosis, transcriptomics

## Abstract

In horizontally transmitted symbioses, structural, biochemical, and molecular features both facilitate host colonization by specific symbionts and mediate their persistent carriage. In the association between the squid *Euprymna scolopes* and its luminous bacterial partner Vibrio fischeri, the symbionts interact with two epithelial fields; they interact (i) transiently with the superficial ciliated field that potentiates colonization and regresses within days of colonization and (ii) persistently with the cells that line the internal crypts, whose ultrastructure changes in response to the symbionts. Development of the association creates conditions that promote the symbiotic partner over the lifetime of the host. To determine whether light organ maturation requires continuous interactions with V. fischeri or only the signaling that occurs during its initiation, we compared 4-week-old squid that were uncolonized with those colonized either persistently by wild-type V. fischeri or transiently by a V. fischeri mutant that triggers early events in morphogenesis but does not persist. Microscopic analysis of the light organs showed that, while morphogenesis of the superficial ciliated field is greatly accelerated by V. fischeri colonization, its eventual outcome is largely independent of colonization state. In contrast, the symbiont-induced changes in crypt cell shape require persistent host-symbiont interaction, reflected in the similarity between uncolonized and transiently colonized animals. Transcriptomic analyses reflected the microscopy results; host gene expression at 4 weeks was due primarily to the persistent interactions of host and symbiont cells. Further, the transcriptomic signature of specific pathways reflected the daily rhythm of symbiont release and regrowth and required the presence of the symbionts.

**IMPORTANCE** A long-term relationship between symbiotic partners is often characterized by development and maturation of host structures that harbor the symbiont cells over the host’s lifetime. To understand the mechanisms involved in symbiosis maintenance more fully, we studied the mature bobtail squid, whose light-emitting organ, under experimental conditions, can be transiently or persistently colonized by Vibrio fischeri or remain uncolonized. Superficial anatomical changes in the organ were largely independent of symbiosis. However, both the microanatomy of cells with which symbionts interact and the patterns of gene expression in the mature animal were due principally to the persistent interactions of host and symbiont cells rather than to a response to early colonization events. Further, the characteristic pronounced daily rhythm on the host transcriptome required persistent V. fischeri colonization of the organ. This experimental study provides a window into how persistent symbiotic colonization influences the form and function of host animal tissues.

## Abstract (precis)

A long-term relationship between symbiotic partners is often characterized by development and maturation of host structures that harbor the symbiont cells over the host’s lifetime. To understand the mechanisms involved in symbiosis maintenance more fully, we studied the mature bobtail squid, whose light-emitting organ, under experimental conditions, can be transiently or persistently colonized by Vibrio fischeri or remain uncolonized. Superficial anatomical changes in the organ were largely independent of symbiosis. However, both the microanatomy of cells with which symbionts interact and the patterns of gene expression in the mature animal were due principally to the persistent interactions of host and symbiont cells rather than to a response to early colonization events. Further, the characteristic pronounced daily rhythm on the host transcriptome required persistent V. fischeri colonization of the organ. This experimental study provides a window into how persistent symbiotic colonization influences the form and function of host animal tissues.

## INTRODUCTION

Symbionts that are acquired each generation by the host from its environment are subject to dramatic changes in ambient conditions as they transition from a free-living to a symbiotic state. Shifts in abiotic factors, such as temperature, pH, nutrients, or osmolarity, as well as biotic conditions, such as cooperation and competition with diverse organisms, constitute major ecological barriers for the survival, replication, and transmission of symbionts. To both adapt to these environmental shifts and grow and persist in the host, bacterial symbionts respond by adjusting gene regulation and expressing specific colonization factors (see, for example references 1 to 4, and for reviews, see references 5 to 7). Similarly, host tissues must recognize and accommodate specific bacterial species and adapt their morphology, physiology, metabolism, and immune regulation to the symbiotic colonization. This adaptation is driven by changes in gene expression during initiation of the symbiosis and later to maintain it (1, 8–10). Ultimately, the stability of the host-microbe partnership results from the coconstruction, by both partners, of an ecological niche in which the net benefits are optimized (11, 12).

Many invertebrates have coevolved with horizontally transmitted microbial communities of low species complexity (13, 14). Among these, the binary nature of the symbiosis between the Hawaiian bobtail squid, Euprymna scolopes, and the luminescent bacterium Vibrio fischeri offers an opportunity to decipher host-symbiont communication with the same advantages as those of the legume-rhizobium symbiosis. For example, because V. fischeri provides no required nutrient, the host squid is easily raised without its symbiont (i.e., aposymbiotically [Apo]) (15).

**FIG 1:**
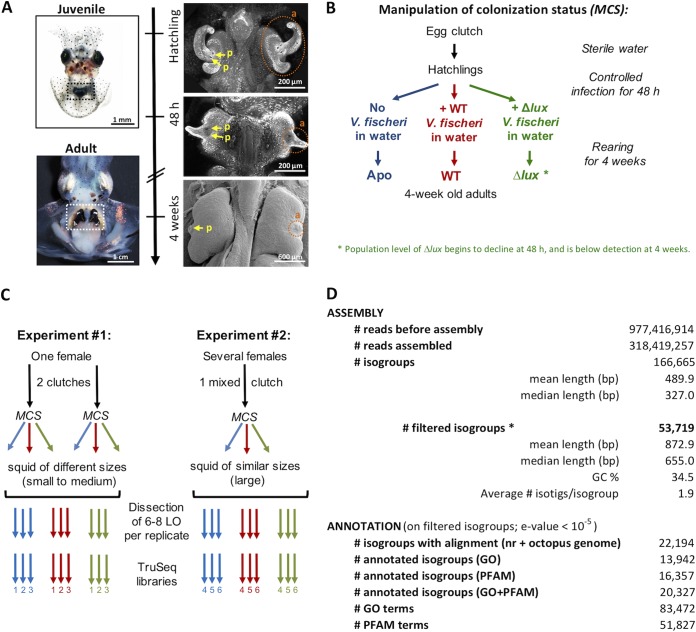
Experimental setup and generation of the light organ transcriptomes of 4-week-old squid. (A) Development of the light organ from hatching (upper left) to adulthood (lower left). The light organ is positioned in the center of the body cavity (dotted rectangles). (Right) Regression of the ciliated appendages (a, orange circles) and the coalescence of the pores (p, yellow arrows) during the 4 weeks postcolonization. (B) Scheme for the manipulation of the colonization status (MCS). Hatchling squid were either not colonized by V. fischeri (Apo) or colonized by wild-type V. fischeri (WT) or by isogenic V. fischeri defective in luminescence production (Δ*lux*). Juveniles were then maintained in separate rearing containers until maturity (4 weeks). (C) Production of RNA libraries. Two experiments were performed sequentially to obtain RNA pools from different egg clutches, with variations for the genetic background and the growth rate. The colonization status of hatchings was manipulated for each clutch, according to the MCS procedure described for panel B. LO, light organ. (D) General statistics on Illumina sequencing, assembly, and annotation. *, filtered isogroups (V. fischeri genes were excluded; size, >240 bp; average expression per condition, >10 reads).

The nascent light organ of the newly hatched squid is prepared to interact with planktonic V. fischeri cells (Fig. 1A) (16, 17), which aggregate on tissue outgrowths, or “appendages,” of complex mucociliary epithelia on the lateral surfaces of the juvenile light organ, which facilitate colonization (18). After 1 to 2 h, the aggregated symbionts migrate into and colonize crypts deep within the light organ, where only V. fischeri can proliferate (19). At each dawn following colonization, the host expels ∼95% of its symbiont population into the surrounding environment in a process called venting (16). The remaining ∼5% of the symbionts proliferate during the day and provide the luminescence used in the squid’s nightly camouflaging behavior, or counterillumination (20). By 12 h postinfection, microbe-associated molecular patterns (MAMPs), notably peptidoglycan (PGN) and lipopolysaccharide (LPS), released by V. fischeri have triggered apoptotic loss of the appendages’ mucociliary epithelia, which potentiate colonization (21). Certain V. fischeri mutants, such as a Δ*luxCDABE* (Δ*lux*) mutant, fully colonize the light organ but do not persist; i.e., by 48 h, the Δ*lux* mutant colonization level has diminished to 50% and is completely lost over the next few days (22, 23).

The colonization of the light organ by V. fischeri also triggers changes in the internal crypt epithelia with which they directly interact. By 12 h, the symbionts have induced a swelling of these host cells that increases their volume 4-fold (22). In addition, by 12 h, interaction with V. fischeri activates a program of increasing host-cell microvillar density; by 4 days, each symbiont cell is surrounded by microvillar membranes. Unlike with the apoptotic loss of the superficial epithelium, the phenotypes of both cell swelling and increased microvillar density are reversible with the loss of symbiont colonization and return to the microanatomy of the nonsymbiotic animal (24, 25). Recent studies have shown that juveniles exhibit a diel rhythm of remodeling of the interface with the symbionts; as the symbionts are vented, the host cells undergo effacement of the microvilli, which re-form in the hours following venting (26).

The maturation of the symbiosis in E. scolopes is completed by around 4 weeks (23, 27), at which time the animal begins burying in the sand during the day, hunting in the water column at night, and using its bioluminescence (unpublished data). Concurrently, the symbionts begin to exhibit a diel metabolic rhythm, from a pH-neutral physiology during the day to an acidification of the crypts during the night (1, 28). Underlying these behaviors is a pronounced diel rhythm of the transcriptomes of both host and symbiont, as well as of the ultrastructure of the host cells (1); whether the host rhythm depends upon the presence of symbionts has not yet been determined.

Because both aposymbiotic (Apo) and symbiotic animals are easily maintained for experiments over the first 48 h of symbiosis initiation, several characteristics of the communication between squid and symbiont have been revealed during this period (9, 10, 22, 25, 29). However, the influence of symbionts on host development and gene expression during the subsequent maturation has not been reported because the rearing of squid under controlled symbiotic conditions became routine only recently (23). Using this technical advance, we addressed two main questions: (i) how do adult light organs’ morphologies and patterns of gene expression compare between uncolonized (Apo), transiently colonized (by the Δ*lux* mutant), and persistently colonized (by the wild type [WT]) squid and (ii) what biological functions in adult hosts are influenced by the presence of symbiosis, and how do they participate in coconstructing a niche in which symbionts thrive? Our data provide evidence of widespread changes in gene expression in 4-week-old light organs that experience a continuing interaction between host and symbiont.

## RESULTS AND DISCUSSION

### Persistent symbiont colonization is required for normal maturation of light organ epithelia.

**FIG 2:**
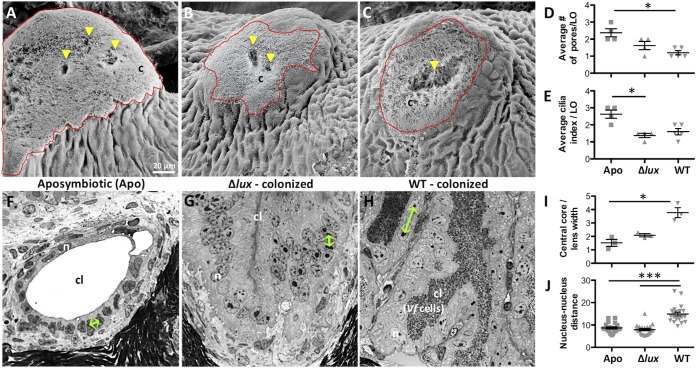
Influence of symbionts on the development and physiology of the light organ. (A to C) Scanning electron microscopy (SEM) images of the superficial ciliated field close to the pore in 4-week-old aposymbiotic (A), Δ*lux* mutant-colonized (B), and WT-colonized (C) squid (to locate the light organ, see the bottom right image in Fig. 1A and the orange circle). The red outline marks the surface covered by cilia (c); yellow arrowheads indicate the locations of pores. The scale bar in panel A is suitable for all images in panels A to C and F to H. (D and E) Statistics on surface structures of the light organ at 4 weeks, namely, the number of visible pores on each side of the light organ (D) and the cilium index, which takes into account the area covered by cilia and the density of long cilia, averaged per light organ (mean ± SE, *n* = 4 or 5; *, *P* < 0.05, Kruskal-Wallis test with Dunn’s multiple-comparison test) (E). (F to H) Images of histological sections through the host crypts of 4-week-old Apo (F), Δ*lux* mutant-colonized (G), and WT-colonized (H) squid. The collapse of the empty crypt lumen (G) is a technical artifact that is independent of the infection status. cl, crypt lumen; n, nucleus; r, reflector; *Vf*, Vibrio fischeri cells. Green arrows mark the representative distances between nuclei under different conditions of colonization. (I to J) Statistics on cellular features of the light organ crypts, namely, the central core width normalized by lens width (mean ± SE, *n* = 3; *, *P* < 0.05, Kruskal-Wallis test with Dunn’s multiple-comparison test) (I) and the distance between nuclei in epithelial cells surrounding the crypt lumen (mean ± SE, *n* = 21; ***, *P* < 0.001 by one-way ANOVA with Tukey’s HSD multiple-comparison test) (J).

We first sought to characterize the anatomical and cellular features of the mature light organ that result from natural, persistent (WT) symbiont colonization and to compare these features to those of light organs that had experienced either a transient (Δ*lux* mutant) or no (Apo) colonization for 4 weeks (Fig. 1B and 2). In general, the surfaces of light organs appeared similar under the three conditions; i.e., the appendages were largely regressed, irrespective of the infection status, with only a small field of cilia remaining visible (Fig. 2A to C). In other studies of maturation (15, 23), some variation in surface features was reported; most notably, the ciliated fields of squid in the Apo condition had regressed to different degrees. This variation is likely due to differences in the tissue’s level of exposure to MAMPs produced by nonsymbiotic environmental bacteria. While the symbiont is uniquely capable of presenting a high concentration of the inductive MAMPs to the crypt tissues ∼12 h after colonization is initiated, constant exposure to low levels of these compounds in the environment can slowly lead to regression (23). Symbiont-produced MAMPs trigger a much more rapid apoptosis-driven regression of the surface ciliated fields (21). In contrast to the similar degrees of regression at 4 weeks, several developmental differences were seen; for example, whereas the pores had typically coalesced in WT-colonized organs (27), the squid in the Apo condition retained the three independent pores of the hatchling state, while the Δ*lux* mutant-colonized organs exhibited an intermediate degree of fusion (Fig. 2D). In addition, the average length and density of epithelial cilia, i.e., the cilium index, was greater in Apo than in Δ*lux* mutant*-* and WT-colonized light organs (Fig. 2E).

To determine whether typical maturation of the crypt epithelia requires persistent symbiont colonization, we sectioned light organs from 4-week-old squid under the three colonization states and measured their cellular dimensions (Fig. 2F to H). To normalize the measurements, we used the width of the light organ lens; this transparent tissue covers the ventral surface of the organ and matures independently of the colonization state (Fig. 2I) (27). The cells of the crypt epithelium of mature light organs colonized by WT V. fischeri were irregularly spaced and exhibited a cuboidal shape; in contrast, the cells of Apo and Δ*lux* mutant-colonized animals were regularly spaced columnar cells that had a lower volume (Fig. 2F to H). This difference was quantified by measuring the nucleus-to-nucleus distance, which in WT-colonized animals was about 1.5 times that of either Apo or Δ*lux* mutant*-*colonized animals (Fig. 2J). These results for mature animals are in concordance with the results of previous studies of symbiosis-induced changes in crypt epithelia of juveniles. In those earlier studies, colonization by WT symbionts for 48 h induced a cuboidal shape and a 4-fold increase in the volume of the crypt epithelial cells, while Apo crypts or those colonized by a dark mutant retained regularly spaced columnar epithelia (22). Taken together, the studies of both early development and maturation provide evidence that the persistent presence of WT bacteria within the crypts is required to retain the changes in crypt epithelial cell shape and volume induced by symbionts.

In summary, some developmental events require the persistent presence of symbionts, e.g., the crypt epithelial phenotype of cell swelling. In contrast, regression of the light organ’s superficial fields of cilia can initiate without colonization if MAMPs are present. Nevertheless, retention of the nature of cilia surrounding the pores of Apo (but not WT- or Δ*lux* mutant-colonized) animals might indicate their capacity to remain open to colonization. In fact, mature light organs cured of their WT symbionts have been shown to lose the ability to be subsequently colonized, while Apo animals or those transiently colonized by the Δ*lux* mutant have not (23). Finally, the loss of the ciliated surface by 4 weeks leaves the crypt epithelium as the major tissue responding to the presence of symbionts and predicts that this interaction dominates the mature stage of the association.

### The principal signature of the mature-light-organ transcriptome reflects persistent host-symbiont interactions.

To identify features of mature-light-organ gene expression that reflect the natural, persistent interactions of symbionts with host epithelia, we constructed a reference transcriptome for the light organs of 4-week-old squid, using the Illumina TruSeq sequencing technology (1 × 100 bp) (Fig. 1). Libraries used to construct this transcriptome were derived from light organs of two independent experiments under the same three conditions described above: uncolonized (Apo) squid or squid colonized by the WT or Δ*lux* symbiont (Fig. 1B and C). Because of genetic variation among the natural host populations used in these experiments and differences in squid growth rates during their culture, the analyses of transcriptomic change revealed only the most robust modifications that had occurred. For each experiment, three pools of between 6 and 8 light organs were extracted per condition, for a total of 18 libraries (Fig. 1C). The ∼318 million reads (average number of reads per library, 17.7 ± 0.6 million; see the quality controls in Fig. S1 in the supplemental material) were assembled into 166,665 isogroups by the Trinity software (Fig. 1D). To limit noise, we selected ∼1/3 of these isogroups as high quality (i.e., those with a length greater than 240 bp encoding a peptide of >80 amino acids and an average expression level of >10 reads/library) (Fig. 1D and Fig. S2). Any sequences identified by BLAST analysis against the V. fischeri ES114 reference genome (30) were excluded from these high-quality isogroups. Over 40% of these isogroups had a significant BLAST hit against either sequences in the nr database or the *Octopus bimaculoides* transcriptome (31). Among these isogroups, 63% were successfully annotated using gene ontology (GO) and 74% using the PFAM database. On average, 4.1 GO terms and 2.6 PFAM terms were assigned per BLAST analysis-annotated isogroup.

10.1128/mSystems.00165-18.1 FIG S1 (A to D) Quality control analyses of RNA-seq libraries, performed by the R package function arrayqualitymetrics (62). (A) Between-library comparison. False-color heatmap of the distances between libraries. Patterns in this plot indicate clustering of the libraries either because of biological factors (Apo, WT-infected, Δ*lux* mutant-infected squid) or unintended experimental factors (experiment 1 or 2). The distance between two libraries is computed as the mean absolute difference between the data of the libraries. No library was detected as an outlier. (B) Variance mean dependence. Density plot of the standard deviations of the intensities across libraries on the *y* axis versus the rank of their means on the *x* axis. The red dots show the running median of the standard deviation. After normalization and transformation to a logarithm(-like) scale, the red line is approximately horizontal; that is, it shows no substantial trend. (C) Library intensity distribution. Density estimates (smoothed histograms) of the data. In this study, the distributions of the libraries have similar shapes and ranges. (D) Individual library quality. MA plots visualize the differences between measurements taken in the library studied and in a pseudolibrary that consists of the median across libraries (M = log ratio, A = mean average). The mass of the distribution in an MA plot is concentrated along the M axis (M = 0), and there is no trend in M as a function of A, suggesting that libraries have similar background intensities and are not saturated. No library was detected as an outlier by computing Hoeffding’s statistic Da on the joint distribution of A and M for each library. Shown are, first, the 4 libraries with the highest values of Da and then the 4 libraries with the lowest values. The value of Da is shown in the panel headings. (E) Validation of the Illumina-normalized counts. The expression of 8 isogroups was measured by qRT-PCR, starting from the total RNA extracts initially used to prepare the libraries, and compared to Illumina-normalized counts. qRT procedures were similar to those described in the article. Candidate gene expression was normalized by taking the geometric mean of the expression of two housekeeping genes (40S ribosomal protein S19 and serine hydroxymethyltransferase [serine HMT]). Six replicates were used per condition, and values are means ± SEs. Download FIG S1, PDF file, 0.6 MB. Copyright © 2018 Kremer et al. 2018 Kremer et al. This content is distributed under the terms of the Creative Commons Attribution 4.0 International license.

10.1128/mSystems.00165-18.2 FIG S2 Statistics on transcript assembly and annotation choice of threshold parameters. (A to B) Choice of threshold parameters for the analysis of isogroups assembled on the basis of their size distribution (A) and their read coverage (B). (C) Comparison between BLAST results for filtered isogroups against sequences in the nr database and against the *Octopus bimaculoides* transcriptome (31). Venn diagrams indicating the number of annotated isogroups showing a BLAST result against the nr database and/or the O. bimaculoides transcriptome (E value threshold = 10^−5^), as well as their annotation with GO and PFAM terms, are shown in the right panels. (D) Sequence similarity (left) and E value distribution (right) from the BLAST results of filtered isogroups against sequences in the nr database and against the *Octopus bimaculoides* transcriptome (31). (E) Species distribution from the BLAST search of filtered isogroups against sequences in the nr database. Download FIG S2, PDF file, 0.8 MB. Copyright © 2018 Kremer et al. 2018 Kremer et al. This content is distributed under the terms of the Creative Commons Attribution 4.0 International license.

The small percentage (41%) of annotated genes agrees with a previous transcriptome analysis of *E. scolopes* (42%) (10) and may result from several factors. First, the specimens were reared from natural populations, where polymorphism and/or degree of RNA editing may be high (31, 32), features that complicate the assembly process. Second, the *O. bimaculoides* genome is the sole cephalopod genome currently available (31). Only 23% of its 38,585 transcripts possess a GO annotation; thus, numerous conserved genes require further investigation. Finally, 10% of the isogroups having a positive BLAST result, 26% of the isogroups having a GO annotation, and 28% of the isogroups having a PFAM annotation were found only when the search was performed against the *O. bimaculoides* transcriptome and not against the nr database (Fig. S2C). These results suggest that the quality of the annotation will rapidly increase once additional cephalopod transcriptomes are released and functionally annotated. Taken together, the data demonstrate that, although only a small fraction of isotigs were annotated by BLAST, they were deeply annotated by GO and PFAM and could be used with confidence in the following transcriptomic analyses.

**FIG 3:**
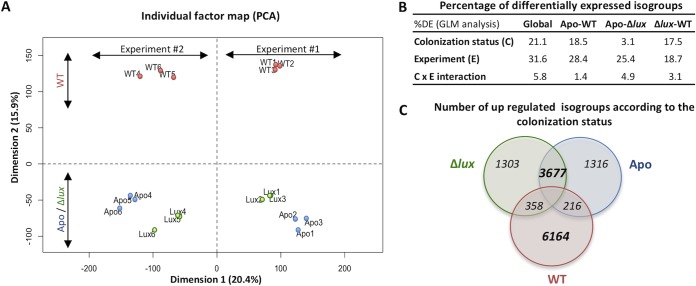
Influence of colonization status and experimental replication on gene expression patterns. (A) Principal-component analysis (PCA) results generated with the variance-stabilized data of the 53,719 filtered isogroups. (B) Percentage of differentially expressed (DE) isogroups after a generalized linear model (GLM) analysis on the whole data set or on subsets containing Apo and WT-colonized, Apo and Δ*lux* mutant-colonized, or WT-colonized and Δ*lux* mutant-colonized squid. We tested whether normalized count data depended on colonization status and experimental replication, and whether there was any interaction between these two factors. (C) Venn diagram of isogroups that were overrepresented after the GLM analyses presented in panel B. In particular, 3,677 isogroups (i.e., 68.9% of the isogroups overexpressed in Apo and 70.6% of those overexpressed in Δ*lux* mutant-colonized light organs) are overexpressed in Apo light organs compared to WT-colonized light organs and overexpressed in Δ*lux* mutant-colonized light organs compared to WT-colonized light organs. In contrast, 6,164 isogroups (i.e., 90.3% of the isogroups overexpressed in WT-colonized light organs) are specifically overexpressed in WT-colonized light organs compared to Apo or Δ*lux* mutant-colonized light organs; i.e., they are not overexpressed in Apo light organs compared to Δ*lux* mutant-colonized light organs or in Δ*lux* mutant-colonized light organs compared to Apo light organs.

To characterize the influence of colonization status on gene expression of mature light organs, we analyzed the normalized count data of the 53,719 isogroups, using a generalized linear model (GLM) that took into account the colonization status (i.e., colonized with the WT or Δ*lux* mutant or uncolonized [Apo condition]) and any experiment-dependent effect (i.e., experiment 1 or 2). The principal-component analysis (PCA), performed on variance-stabilized, transformed dispersion data, indicated that the colonization status and the experiment number explained ∼16% and 20% of the variances, respectively (Fig. 3A). Overall, 11,328 isogroups were statistically differentially expressed in response to a change in the colonization status. Although the two experiments were associated with different expression profiles, the three replicates from each colonization status always grouped together, and only a negligible interaction was observed between the colonization status and the experiment (Fig. 3A and B). Around 70% of the isogroups that were differentially expressed between Apo light organs and WT-colonized light organs were also differentially expressed between light organs colonized by the Δ*lux* mutant and light organs colonized by the WT. In contrast, 90.3% of the isogroups that were differentially expressed between WT-colonized light organs and Δ*lux* mutant-colonized or Apo light organs were not differentially expressed between Δ*lux* mutant-colonized and Apo light organs (Fig. 3C). These results indicate that symbiotic light organs exhibit a pattern of expression different from that of both aposymbiotic organs and organs only transiently colonized by the Δ*lux* mutant. Thus, the data provide evidence that the presence of symbionts was the single most important determinant in the signature of the light organ transcriptome. The Apo and Δ*lux* mutant colonization conditions exhibited comparable patterns of expression, which may reflect the similarities observed in the structures of crypt epithelial cells (Fig. 2F to J). Taken together, these results suggest that lasting changes in gene expression associated with any developmental events irreversibly induced by a transient colonization are minor compared to the direct effect of a persistent interaction of the symbionts in the mature light organ.

### Transcriptional changes shared between mature WT- and Δ*lux* mutant-colonized animals reflect processes associated with the initial stages of the symbiosis.

The patterns of the transcriptomic data described above support the conclusion that the crypt epithelium numerically dominates the epithelial surface in setting the transcriptomic signature of the mature, colonized light organ. However, our experimental conditions, specifically WT and Δ*lux* mutant colonization, provided an opportunity to explore what portion of the mature transcriptome results from the initial irreversible developmental signal(s) induced by either WT or Δ*lux* mutant colonization (21, 33). Further, our experimental conditions allow analysis of these results in comparison to the smaller differences in tissue regression patterns observed in the light organ’s superficial structures of Apo animals. Using a generalized linear-model analysis on normalized count data, we defined isogroups that were statistically overexpressed between both WT- and Δ*lux* mutant-colonized light organs and Apo light organs (Fig. 3C and Table S1). From these 358 isogroups, we extracted the corresponding terms according to various ontologies and performed an enrichment analysis on those belonging to the three gene ontologies (biological processes, molecular functions, and cellular components, i.e., the most significant [*P* < 10^−4^] GO functions [Table 1; Tables S2 and S3]) and to the PFAM ontology (Table S4). Only the copper ion-binding molecular function was significantly overrepresented in comparison to what was expected (*P* value adjusted for the false-discovery rate [FDR], <10^−4^). However, the PFAM analysis (Table S4) indicated that a few domains, such as those specific to chitinases (GH20 domain), hemocyanin (multicopper oxidase domain), lipopolysaccharide-binding proteins (LBPs), and bactericidal/permeability-increasing proteins (BPIs), already known to play a role in the establishment of the symbiosis (10, 34, 35), were overrepresented in both WT- and Δ*lux* mutant-colonized light organs, compared to Apo light organs. For instance, the increased expression of *E. scolopes* LBP1 in WT-colonized crypts has been implicated in signaling to the host during MAMP-induced regression of the superficial ciliated epithelium (35).

**TABLE 1:**
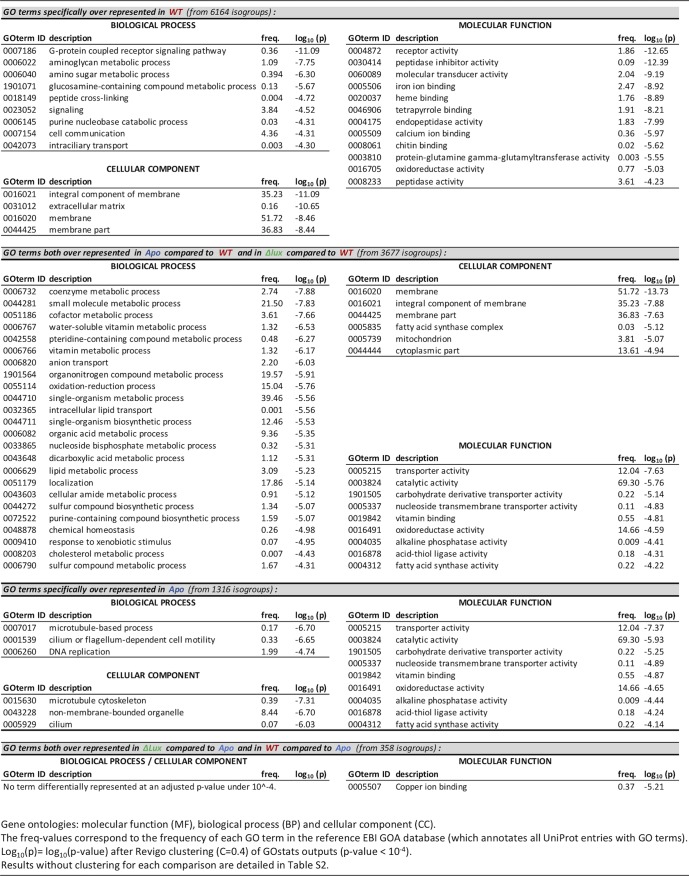
Functional enrichment analysis based on gene ontology

10.1128/mSystems.00165-18.3 TABLE S1 Differential expression of isogroups in response to infection. List of isogroups differentially represented after a generalized linear model analysis of the 53,719 filtered isogroups. Normalized count numbers are indicated for the 6 replicates of each modality (experiment 1 consisted of replicates 1, 2, and 3; experiment 2 consisted of replicates 4, 5, and 6). The GLM analysis on normalized count data (infection status and experiment used as fixed factors) was here performed on the whole data set. The significance of each factor (infection, experiment, interaction between infection and experiment) is indicated by “true” or “false” (*P*  < 0.05 or *P*  > 0.05, respectively), with the corresponding log_2_ fold change. The last sheets (names starting with VD) provide the lists of isogroups from each section of the Venn diagram (Fig. 3C). These isogroups were used for the GOstats and REViGO analyses. Download Table S1, XLSX file, 4.1 MB. Copyright © 2018 Kremer et al. 2018 Kremer et al. This content is distributed under the terms of the Creative Commons Attribution 4.0 International license.

10.1128/mSystems.00165-18.4 TABLE S2 Functional enrichment analysis based on the gene ontology (GOstats output). List of GO terms differentially represented in response to infection. Hypergeometric tests have been performed from lists of differentially expressed (DE) isogroups using the GOstats Bioconductor package, and *P* values have been corrected for multiple testing using an FDR correction. GO terms in red are those that have a corrected *P* value of <0.01 and were used for the REViGO analysis (Table S3). BP, biological process; MF, molecular function; CC, cellular component; Spe_over, specifically overrepresented (under a particular symbiotic condition). Download Table S2, XLSX file, 0.2 MB. Copyright © 2018 Kremer et al. 2018 Kremer et al. This content is distributed under the terms of the Creative Commons Attribution 4.0 International license.

10.1128/mSystems.00165-18.5 TABLE S3 Functional enrichment analysis based on the gene ontology (REViGO output). List of clustered GO terms differentially represented in response to infection. GO terms identified by GOstats (Table S2) and whose corrected *P* value was <0.01 were used as input for REViGO analysis (simrel, *C* = 0.4). GO terms in red are those that have a *P* value of <10^−4^ and are reported in Table 1. Spe_over, specifically overrepresented (under a particular symbiotic condition). Download Table S3, XLSX file, 0.04 MB. Copyright © 2018 Kremer et al. 2018 Kremer et al. This content is distributed under the terms of the Creative Commons Attribution 4.0 International license.

10.1128/mSystems.00165-18.6 TABLE S4 Functional enrichment analysis based on the PFAM ontology. List of PFAM terms differentially represented in response to infection. Hypergeometric tests have been performed from lists of DE isogroups using the GOstats Bioconductor package, and *P* values have been corrected for multiple testing using an FDR correction. Spe_over, specifically overrepresented (under a particular symbiotic condition). Download Table S4, XLSX file, 0.05 MB. Copyright © 2018 Kremer et al. 2018 Kremer et al. This content is distributed under the terms of the Creative Commons Attribution 4.0 International license.

Genes were also identified in the 1,316 isogroups that were irreversibly turned down in both WT- and Δ*lux* mutant-colonized light organs but not in Apo light organs (i.e., specifically turned up in Apo light organs) (Table 1). These signatures represent cilium motility and a microtubule-based process, which were downregulated by an early symbiosis event. PFAM analysis reinforced this signature by identifying an overrepresentation of domains involved in dynein motors and potential antiapoptotic domains, such as the NACHT domain. Together, these functions are consistent with early, irreversible symbiosis-induced events like regression of the ciliated surface epithelium. These loci shared by WT- and Δ*lux* mutant-colonized mature light organs represent a few genes whose expression is irreversibly changed by the first 24 to 48 h of colonization.

### Transcriptional changes unique to mature WT-colonized light organs indicate an impact on specific metabolic processes and tissue homeostasis.

Whereas the initiation of symbiosis by either WT or *Δlux* symbionts mediates early light organ development (e.g., ciliated-field regression), only the persistent colonization by the WT evokes an effect on both cell morphology and the transcriptome that reflects events in the crypt epithelium (Table 1 and Tables S1 to S4). For example, the 6,164 isogroups that were overrepresented only in WT-colonized organs revealed a major transcriptomic signature related to cell-cell signaling (Table 1). These results suggest that the mature crypt epithelium actively and continuously responds to symbiont signals, a trend confirmed by the PFAM analysis (Table S4). Indeed, the upregulation of domains associated with receptor family ligand binding, lectin, the TIR domain, or the immunoglobulin domain suggests a response characteristic of those previously associated with host-bacterial interactions (36).

Focusing on isogroups either downregulated by symbiosis or upregulated in the absence of symbionts (i.e., overexpressed in both Apo and Δ*lux* mutant-colonized light organs compared to WT-colonized light organs) revealed enrichment for processes such as transmembrane transport and chemical homeostasis, including anion transport and lipid transport (Table 1). These transcriptomic signatures are consistent with the idea that crypt cell swelling associated with symbiotic organs is linked to fluid uptake. Specifically, regulation occurred in genes controlling osmotic regulation, notably a Na^+^-K^+^-Ca^2+^ exchanger, organic-anion exchanger, atrial natriuretic peptide-converting enzyme, and sialin (Table S1). Expression was also modified in transcripts that encode proteins involved in the metabolism of coenzymes, cofactors, vitamins, and lipids (Table 1). Remarkably, the oxidoreductase activity was overrepresented in all comparisons, although the specific isogroups were different in that they were shared by Apo and Δ*lux* mutant-colonized light organs but different from those of WT-colonized light organs. Thus, a modulation of oxidoreductase activity appears to be characteristic for all states, i.e., in the presence or absence of symbionts.

### In the mature association, symbionts influence the expression of the specific host gene on a daily rhythm.

**FIG 4:**
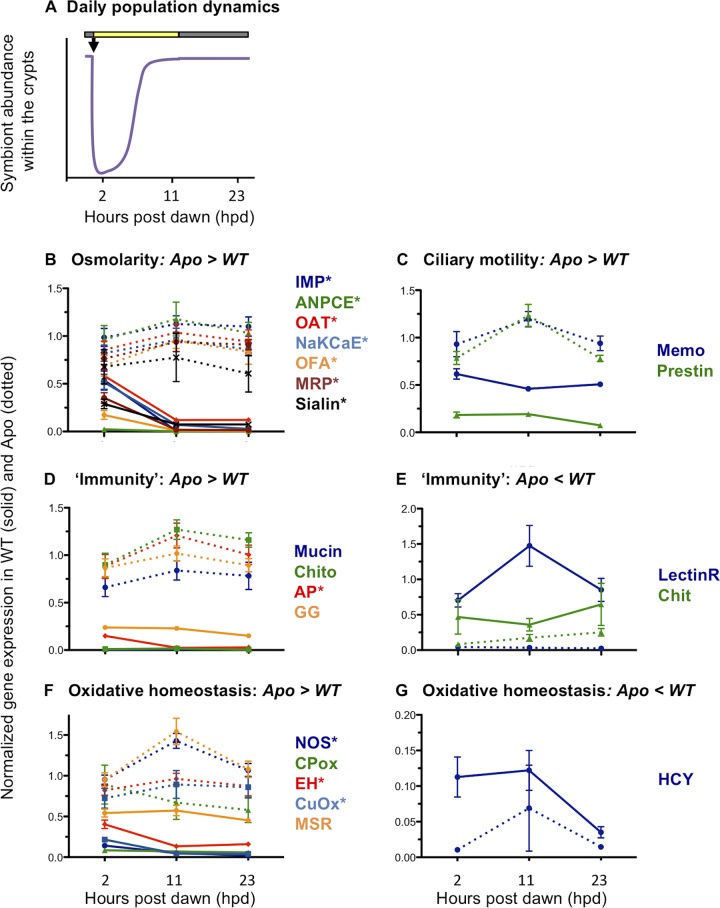
Influence of diel rhythm on host gene expression. (A) Fluctuation of bacterial abundances over the day/night cycle. Ninety-five percent of the symbiont population is expelled at dawn (black arrow). The remaining population grows exponentially to reach a plateau around 6 h postdawn (hpd). (B to G) Differential expression of candidate genes at 3 time points over the daily cycle (2, 11, and 23 hpd). These genes are involved in osmotic regulation (B), ciliary motility (C), immunity (D and E), and oxidative homeostasis (F and G). The qRT-PCR expression data from candidate genes were normalized by the expression of two housekeeping genes, serine hydroxymethyltransferase and 40S ribosomal protein S19. Values are means ± SEs (5 replicates per condition). Genes followed by an asterisk are those whose expression in WT-colonized squid is significantly different at 2 hpd, i.e., when the bacterial density is low, from their expression at the two other time points (11 and 23 hpd), when bacterial density is high. Statistical analyses were performed by ANOVA of log-transformed data, followed by the Tukey HSD test (see Table S5 for BLAST annotation and Table S6 for all statistical analyses). Annotation of candidate genes results from BLAST searches against sequences in the nonredundant (nr) database. IMP, inositol monophosphatase; ANPCE, atrial-natriuretic-peptide-converting enzyme; OAT, organic anion transporter; NaKCaE, Na-K-Ca exchanger; OFA, oxalate-formate antiporter; MRP, multidrug resistance protein; Memo, mediator of cell motility; Chito, chitotriosidase; AP, alkaline phosphatase; GG, Golden Goal; LectinR, lectin receptor; Chit, chitinase; NOS, nitric oxide synthase; CPox, chorion peroxidase; EH, epoxide hydrolase; CuOx, copper oxidase; MSR, methionine sulfoxide reductase; HCY, hemocyanin. Note that a few candidate genes were not differentially expressed by qRT-PCR, using extracts from this new cohort, although these genes were overexpressed in symbiotic light organs compared to aposymbiotic ones in the transcriptome sequencing (RNA-seq) experiment. The proteins for these genes include the following: duox, FAD oxidoreductase, ferritin, glutathione *S*-transferase (GST) omega, cadherin, cathepsin L, chitin synthase, LBP1, BPI3, and a metalloprotease. This absence of differential expression might be explained by (i) genetic differences in the two cohorts, (ii) a low differential expression in the RNA-seq experiment, or (iii) developmental differences.

Symbiont abundance in the crypts undergoes dramatic changes over a daily cycle, with a maximal density (i.e., number of bacterial cells/light organ) during the night and a minimum density at dawn, after the venting of 95% of the symbionts (Fig. 4A) (16). Global transcriptomic analyses showed that at 11 h postdawn (hpd), when the symbiotic density is highest and the apical-basal polarity of the host epithelia is most pronounced (1, 26), maintenance of symbiosis was linked to a downregulation of genes involved in specific metabolic and chemical homeostasis pathways (Fig. 3, Table 1) that may have a direct impact on cell physiology and morphology. To confirm that the presence of symbionts directly drives the expression of these genes, we took advantage of the natural diel rhythm of symbiont abundance within the crypts; specifically, we chose a few representative candidate genes (Table S5) and compared their levels of expression in Apo and WT-colonized light organs at three different time points over the daily cycle, i.e., 2, 11, and 23 hpd (see the details of the statistical analyses in Table S6). If the transcriptomic response is linked directly to symbiont density (or indirectly, through a host diel rhythm entrained by the symbionts), we would expect patterns of gene expression to be more similar between Apo and WT-colonized light organs immediately after venting (i.e., 2 hpd) than when bacterial density was high (i.e., 11 and 23 hpd). When a diel cycle was observed in WT-colonized animals, we controlled for the possibility that the gene expression pattern was not intrinsic to the system (e.g., an entrained circadian rhythm). Indeed, around the venting period, Apo light organs exhibited either no pattern or a pattern of expression opposite to that of WT-colonized light organs (Fig. 4).

10.1128/mSystems.00165-18.7 TABLE S5 Sets of primers used in this study and efficiency of RT-PCRs. Download Table S5, XLSX file, 0.04 MB. Copyright © 2018 Kremer et al. 2018 Kremer et al. This content is distributed under the terms of the Creative Commons Attribution 4.0 International license.

10.1128/mSystems.00165-18.8 TABLE S6 Statistical analysis of changes in light organ gene expression in response to symbiosis, over the day/night cycle. Download Table S6, XLSX file, 0.03 MB. Copyright © 2018 Kremer et al. 2018 Kremer et al. This content is distributed under the terms of the Creative Commons Attribution 4.0 International license.

Our first focus was on the rhythmic activities of genes potentially involved in maintaining cellular features of the mature crypt epithelia (e.g., osmolarity and ciliary motility) (Fig. 4B and C; Table S6). Analysis of another cohort of 4-week-old squid confirmed the overexpression of these genes in Apo light organs compared to WT-colonized light organs. Interestingly, the expression of essentially all those genes involved in osmotic regulation (Fig. 4B) significantly increased after venting (2 hpd), when the bacterial density drops, and decreased again after bacterial repopulation in the crypts (11 hpd). This pattern is unlikely to be simply a diel response, as it does not appear in Apo light organs (Fig. 4B). Taken together, these data reinforce the idea that the host perceives the changing symbiont density directly through a change in the levels of either MAMPs (e.g., LPS or PGN) or metabolic products (1, 28) excreted by the symbionts and adjusts the expression of its genes accordingly. Osmotic regulation may, thus, be a dynamic process of the crypt epithelium and strongly depend on the presence of symbionts. In contrast, the expression of genes involved in ciliary motility (Fig. 4C) did not vary coordinately with the daily cycle in WT-colonized light organs, which suggests that, even with the loss of the ciliary surface, ciliary motility is a conservative activity of the symbiotic state and does not require the presence of symbionts.

We next analyzed genes involved in host physiology that might play a role in the accommodation, and/or maintenance, of the symbionts and their activity within the mature light organ. We selected candidate genes involved in either (i) immunity, as broadly defined (i.e., adhesion, recognition, or colonization), or (ii) oxidative homeostasis (Fig. 4D to G; Table S6). Except with alkaline phosphatase, the levels of transcription of the six identified immunity genes did not exhibit a significant difference across the daily time points (Fig. 4D and E), suggesting that even the presence of 5% of the symbiont population was sufficient to maintain their expression. In contrast, the expression of half of the genes encoding oxidative homeostasis proteins (e.g., NO synthase, copper oxidase, and epoxide hydrolase) was highest after venting (2 hpd) and decreased again after bacterial repopulation in the crypts (11 and 23 hpd) (Fig. 4F and G). While these genes encode both pro- and antioxidant activities, their lower expression in colonized light organs suggests that the presence of symbionts impacts the oxidative environment and/or its regulation (37, 38). An exception was hemocyanin, a critical component of oxygen transport and light emission during the night (28, 34), whose gene was most expressed in symbiotic light organs (Fig. 4G).

### Continuous interaction between partners favors the maintenance of the symbiotic niche and affects coevolutionary processes.

**FIG 5:**
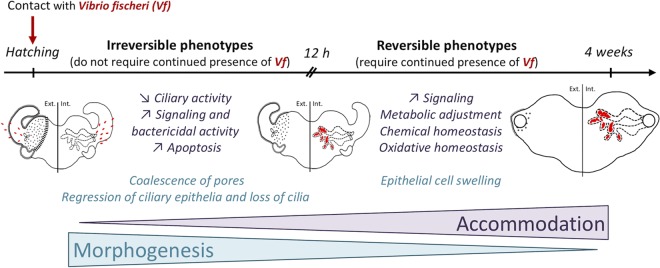
Model of squid-vibrio interactions. V. fischeri (red) initially induces an irreversible morphogenetic signal that shapes the light organ. The presence of the symbiont then actively influences host genes, whose expression changes to accommodate symbionts and their long-term maintenance within the light organ. The light organ was drawn by Suzanne Peyer and is reproduced with her permission. Ext., external; Int, internal.

A key question in symbiosis is “how are beneficial microbes recognized and tolerated by the host, while pathogens are eliminated?” One well-described selected strategy is the development of specialized host structures, such as the bacteriome of grain weevils, in which the immune response is modulated and permits the maintenance of the symbiont (39). In the mature-squid light organ, while the presence of WT symbionts promoted certain signaling cascades, no typical immune pathways were upregulated relative to those of Apo or Δ*lux* mutant-colonized squid (Table 1). For instance, in one cohort of animals, changes in oxidative-stress-related genes generally involved in the acute stress response (e.g., dual oxidase and ferritin [unpublished data]) were either low or undetectable. These results suggest that, after 4 weeks, the host has accommodated itself to the presence of symbionts (Fig. 5), much as plants do after colonization by mycorrhiza. Specifically, while many plant stress genes that respond to either pathogens or oxidative damage are upregulated during the initial infection process, their expression moderates as the symbiosis matures (e.g., during root nodule formation) (40, 41). Whether a similar level of accommodation occurs in the maturing light organ is still unknown; however, it is suggestive that hemocytes isolated from mature, but not juvenile, animals specifically tolerate V. fischeri, reducing their phagocytic activity toward the symbionts (42).

Long-term interactions between symbiotic partners can drive a coevolutionary adaptation of the host to the presence of symbionts. In extreme circumstances, molecular mechanisms involved in these adaptations, such as the regulation of the oxidative environment, eventually lead to dependence of the host on its symbiont (37). In the coevolved association between the Hawaiian bobtail squid and V. fischeri, bioinformatic analyses that reveal biological functions contingent on the mature symbiosis can suggest how selection has driven the host’s accommodation to its symbiont. For instance, although more isogroups were upregulated in persistently WT-colonized light organs compared to the number of upregulated isogroups shared in Apo and transiently Δ*lux*-colonized organs, only a few biological processes were specifically overrepresented (37 processes for 6,164 isogroups; GOstats *P* value adjusted for the FDR, <0.01) compared to the number of overrepresented processes shared by Apo and transiently Δ*lux* mutant-colonized organs (202 processes for 3,677 isogroups). These results indicate that the presence of symbionts targets specific processes, whereas their absence derepresses a number of functions involved in a range physiological processes. In addition, while a few global GO terms can include both inducing and repressing activities, the transcriptomic data suggest that the presence of symbionts within the light organ generally results in the repression of host activities antithetical to the production of an immunological and metabolic environment in which they can thrive. In contrast, the transcriptomic pattern of Apo animals may reflect the absence of normal symbiosis-induced development within the light organ, resulting in a perturbation of cellular homeostasis (Fig. 5); that is, over evolutionary time, the host has become reliant on interactions with its symbiont. The apparent dysbiosis arising in the absence of symbionts is an outcome not unlike the onset of human autoimmune disease hypothesized to result from a reduced microbiome diversity, i.e., the hygiene hypothesis (43).

### Conclusions.

Our studies of the mature squid-vibrio symbiosis provide a window into the impact of persistent partner interaction on both tissue form and function and associated host gene expression. The binary nature of this natural model system offers the opportunity to experimentally control the bacterial side of the partnership; specifically, the morphological and transcriptomic responses of the host that we report are due to the presence (transient or persistent) or absence of a single bacterial species. In addition, while the host developmental trajectory is irreversible, there is no permanent accommodation of the symbiont to host tissues. Because 95% of the symbiont population returns to a planktonic existence each day, with little likelihood of initiating another symbiosis, each cell retains its adaptation to a free-living niche. Thus, the squid host neither induces a bacteroid state, as in certain root nodules (44), nor has a successional development of its microbiota over ontogeny, as is characteristic of mammals (45). In this first experimental study of maturation, we have shown that early, transient exposure to the symbiont, at a time when significant tissue morphogenesis occurs, did not persistently imprint upon the gene expression of the mature organ. In future studies, to further dissect the mechanisms driving these patterns of host response, we will colonize with V. fischeri mutants that either persist until maturity before being lost (28) or persistently colonize the organ but at only a fraction of the level of their wild-type parent (46). Together, these studies will further define the principles underlying the dependence of host development on its microbiota.

## MATERIALS AND METHODS

### General procedures and rearing conditions.

Vibrio fischeri strains (wild-type ES114 [47] and the derived ES114 *ΔluxCDABEG* [Δ*lux*] mutant, which is defective in light production [48]) were grown in LBS medium (23). Mature Hawaiian bobtail squid (*Euprymna scolopes*) were collected in Hawaii (Maunalua Bay, Oahu Island) and transported to the University of Wisconsin—Madison, where they were maintained in a recirculating artificial seawater (Instant Ocean; IO) system. All experiments conform to the relevant regulatory standards established by the University of Wisconsin—Madison. Egg clutches derived from this breeding colony were individually maintained at 23°C (23). For each experiment (Fig. 1B), juveniles were collected and placed in 100 ml of filter-sterilized IO containing either no V. fischeri organisms (i.e., aposymbiotic [Apo]), 5,000 CFU/ml of wild-type V. fischeri (i.e., symbiotic [WT]), or 5,000 CFU/ml of Δ*lux* V. fischeri. After 24 h and 48 h of inoculation with/without bacteria and every week until sacrifice, the infection status was checked by measuring the luminescence emission with a Turner TD-20/20 luminometer (Turner Design); Apo and Δ*lux* mutant-colonized squid remained dark, whereas WT-colonized squid were luminescent. To confirm that Δ*lux* squid were indeed colonized, a few juvenile squid were checked at 24 h and 48 h postinoculation by scoring bacterial colonies on LBS plates from squid homogenized in modified phosphate-buffered saline (mPBS). In addition, the release of Δ*lux* bacteria was checked by PCR after a water change using HvnC primers for V. fischeri detection (49) and 27F/1492R primers for a 16S rRNA-positive amplification control (50). Animals were maintained for 4 weeks (see reference 23) on a 12 h/12 h light/dark (L/D) cycle; within 1 h after the onset of light, the venting process, i.e., expulsion of ∼95% of the bacterial population into the seawater, occurred. Because the light organ symbiosis is mature and the transition to adult diel behavior, i.e., active hunting behavior during the night and quiescence during the day, occurs at around 3 to 4 weeks of age (E. J. Koch, personal observation), we sampled the animals at 4 weeks posthatching.

For the Illumina sequencing experiment and microscopic characterizations, we sampled squid at 11 h postdawn (hpd), i.e., just before dusk, when the bacterial density is maximal and the crypt epithelia are fully polarized (1). The experiment was performed twice, but the two batches slightly varied in terms of the genetic pool (the number of females participating in the egg clutch) and growth rate (Fig. 1C). In the first experiment, we used juveniles from different clutches (L108 and L117) laid by the same female, and we equally pooled the individuals from the two clutches for the collection. Although sampled squid were all 4 weeks old, they were heterogeneous in size (23% were small, 56% were medium, and 14% were large; respective mantle lengths were 3 mm, 4 mm, and 5 to 6 mm). In the second experiment, we used juveniles from one large clutch laid by 1 to 3 females present in the tank during the collection trip. Sampled squid were more homogeneous in size (56% were medium and 44% were large) than in the first experiment. To reduce sampling bias, for the quantitative real-time PCR (qRT-PCR) experiment, we used animals reared from 3 clutches, with each clutch from a different female (replicates 1 and 2, clutch R85; replicate 3, clutch R86; replicate 4, clutch R93; replicate 5, a mix of the 3 clutches; in all cases, the mantle lengths of the animals were >3 mm). The squid were sampled at 3 times during the diel cycle: 2, 11, and 23 hpd.

### Microscopic observation of the light organ.

The morphology and cellular characteristics of light organ cells were monitored at 4 weeks in Apo, WT-colonized, and Δ*lux* mutant-colonized animals (three squid per condition from experiment 2, with sizes varying from small to large). Dissected light organs were prepared for microscopy as previously described (26). One-micrometer-thick sections that had been embedded in Spurr’s resin were cut using an EM UC7 ultramicrotome (Leica), mounted on glass slides, stained with 1% toluidine blue and 1% borate for 30 s, and rinsed with water before visualization on an Axio Imager M2 (Zeiss). The central core/lens width measurement corresponds to the ratio between the widths of two light organ tissues (for localization of the tissues, see Fig. 1A in reference 28): the central core tissue (as defined by the presence of crypt epithelial cells, measured at its widest point in the lateral plane) and the width of the lens (measured at its widest point on the same axis). The nucleus-to-nucleus distance was estimated using two cells of equivalent sizes in the crypt epithelium (to eliminate potential differences due to the plane of section) and by measuring the distance between the center of the nucleus in one cell to the center in the adjoining cell (5 to 6 measurements/animal).

The exterior morphology of the light organ was examined by scanning electron microscopy (SEM) in 4-week-old Apo, WT-colonized, and Δ*lux* mutant-colonized animals (4 to 5 squid/condition from experiment 2, with size varying from small to large), using a Hitachi S-570 LaB6 scanning electron microscope. Samples were prepared as previously described (23), except that the funnel was removed during the 70% ethanol dehydration step; samples were then dried using a Tousimis Samdri 780 critical point drier and coated with gold using a SeeVac Auto Conductavac IV sputter coater (23). The number of visible pores and the cilium index, which takes into account the area covered by cilia and the density of long cilia, were determined in a blind fashion on both sides of each light organ observed (4 to 5 animals/condition).

### Transcriptomic database using Illumina sequencing. (i) RNA extraction and sequencing.

Squid that had been collected at 11 hpd, stabilized overnight at 4°C in RNAlater (Ambion), and frozen at −80°C were used for RNA extraction. Light organs were dissected in RNAlater, and RNA from a pool of 6 to 8 light organs per experimental condition was extracted using the RNeasy kit (Qiagen), with the following modifications to the manufacturer’s instructions. Disruption was performed using a TissueLyser (30 Hz for 4.5 min) followed by a 2-min centrifugation step (13,000 rpm) on a QIAshredder column (Qiagen). RNA was then treated for potential DNA contamination using the Turbo DNase kit (Ambion). The quality of the RNA extracts was checked using a 2100 Bioanalyzer (Agilent), and the quantity of RNA was measured using the Qubit instrument (Life Technology). Libraries were prepared and sequenced by the UW Biotechnology Center, starting from 1 µg of total RNA for the Apo and Δ*lux* mutant conditions and from 1.3 µg of total RNA for the WT condition, to correct for the underestimation of the squid RNA quantity due to the presence of bacterial RNA in the WT samples. All samples were prepared simultaneously using the TruSeq Illumina technology (TruSeq mRNA protocol Rev.A with a 6-min fragmentation step and a ligation step using 18 specific adaptors [1/condition]). Libraries were validated on a BioTek Synergy2 PicoGreen plate reader before being sequenced across 6 lanes (18 samples/lane) on a HiSeq 2000 (1 × 100 bp).

### (ii) *De novo* assembly and mapping.

Initial reads were filtered using FASTX-toolkit (http://hannonlab.cshl.edu/fastx_toolkit/commandline.html) to remove adaptors and low-quality reads (Phred score < 20, coverage > 80%). To obtain a *de novo* transcriptome of 4-week-old squid light organs (i.e., potential transcripts and their isoforms), Trinity software (version 2) was run using a file containing filtered data from all experimental conditions and using parameters defined for single-end sequencing (Trinity version 2013-08-14 [51]). For each condition, reads were mapped on the *de novo* transcriptome using Bowtie (version 2, 2.1.0 [52]), and those that successfully mapped were counted using RSEM (version 1.2 [53]).

### (iii) BLAST and GO/PFAM annotation.

When genes (i.e., isogroups) possessed different isoforms (i.e., isotigs), the longest isoform was selected for further analysis. A selection of biologically relevant isogroups (i.e., filtered isogroups) was performed as follows: (i) only isogroups whose lengths were over 240 bp, i.e., encoding >80 amino acids, and whose mean expression per experimental condition was 10 reads were selected and (ii) all isogroups with BLAST hits in the genome of V. fischeri were discarded (BLASTn E value < 1.0^−50^, GenBank accession number NC_006840.2).

A generalized linear model was then performed on gene expression data from these filtered isogroups (with the DESeq package [R software]), using the normalized count data. The colonization status and the experimental replication were used as fixed factors. The method was set to “pooled,” and the sharingMode was set to “fit-only” (54). A principal-component analysis (PCA) was performed on variance-stabilized transformed dispersion data using the FactoMineR package (R software) (55). To determine isogroups that were differentially expressed between two infection states, a general linear model (GLM) analysis was performed on a subset of the global dispersion data. All isogroups whose infection effect was significant, taking into account potential interactions, were selected and sorted by log_2_ fold change. Only isogroups that were upregulated between two conditions were used for the enrichment analysis.

The filtered isogroups were finally aligned using BLAST (BLASTx, annotation rule cutoff = 55, E value < 10^−5^, hit–high-scoring pair [HSP] overlap = 0, GO weight = 5) against the nonredundant (nr) database (release 2.2.26) and against the transcriptome of *Octopus bimaculoides* (31). xml files derived from the BLAST analysis were imported into the Blast2GO interface, from which terms from the gene ontology (GO) were extracted (56). To define the GO functions that were overrepresented between experimental conditions, we performed hypergeometrical tests (i.e., enrichment analysis) using the GOstats Bioconductor package (the *P* value was set at 1% after the false discovery rate’s correction) (57) (Table S2). This method tests whether the GO terms that were extracted from the list of genes that are upregulated are statistically significantly overrepresented compared to the GO terms extracted from the reference gene list (which corresponds to the entire data set; i.e., 53,719 isogroups). GOstats outputs were then loaded into REViGO software (58) to reduce redundancy within the list of differentially represented GO terms for a specific comparison through a clustering algorithm of similar semantic GO terms (similarity, 0.4; reference, UniProt; semantic similarity, simrel) (Table S3). A similar enrichment analysis was conducted using PFAM annotations using the GOstats Bioconductor package. These PFAM terms were either directly obtained from the *O. bimaculoides* database (31) or extracted from an hmmscan of the isotigs (59) using the Pfam-A.hmm database as the template (TransDecoder/HMMER).

### Expression of candidate genes.

To study the effect of the bacterial density on candidate gene expression, we collected 4-week-old squid from a new cohort of animals at three time points over the day-night cycle (i.e., 2, 11, and 23 hpd; 5 replicates/condition/time point), and stabilized them in RNAlater as described above. Four to five light organs were dissected per replicate, and RNA was prepared similarly to the way the Illumina libraries were prepared. Because of the increased presence of bacterial RNA during the daily population regrowth and based on preliminary RNA quantifications, total RNA quantities (500 µg) were adjusted for cDNA synthesis by a factor of 1.1 for the WT condition at 11 hdp and by a factor of 1.2 at 23 hpd. qRT-PCR procedures conform to the Minimum Information for Publication of Quantitative Real-Time PCR Experiments (MIQE) guidelines (60) and follow already-published protocols (10), with the following specifications. cDNAs were synthetized by the SMART Moloney murine leukemia virus (MMLV) reverse transcriptase (Clontech) from oligo(dT)_12–18_ primers (Life Technologies) and diluted 1/8-fold in water. qPCR mixes, consisting of 0.5 μl of each primer (10 μM), 5 μl of SsoAdvanced SYBR Green supermix (Bio-Rad), and 4 μl of a 1/8 dilution of the cDNA reaction mixture, were run on a CFX Bio-Rad instrument (2 technical replicates/biological replicate) as follows: 3 min at 94°C and 40 cycles of 10 s at 94°C, 10 s at 60°C, and 15 s at 72°C, with a melting curve from 70°C to 95°C. Primer sets (see Table S5) exhibit PCR efficiencies between 92.3 and 101.9 (mean ± standard error [SE] = 97.5 ± 0.33), which were used to calculate expression values based on Pfaffl’s method (61). Expression of each candidate gene was normalized by the geometric mean of the expression of two housekeeping genes (40S ribosomal protein S19 and serine hydroxymethyltransferase [serine HMT]). Expression data were log transformed before we performed an analysis of variance (colonization status and time were used as fixed factors). Two-way analysis of variance (ANOVA) residuals were checked for normality by Shapiro’s test and for homoscedasticity by Levene’s test. Pairwise comparisons were performed using Tukey’s honestly significant difference (HSD) test (R software, R Core team 2015, version 3.2.3).

### Accession number(s).

Sequences were deposited in the Sequence Read Archive under the biological project PRJNA320238.
