# Supplementary material for: Persistent Interactions with Bacterial Symbionts Direct Mature-Host Cell Morphology and Gene Expression in the Squid-Vibrio Symbiosis
Source: mSystems. 2018 Oct 2;3(5):e00165-18. doi: 10.1128/mSystems.00165-18 (PMC6172772; doi:10.1128/mSystems.00165-18)

Supplementary file 1

Library	Name	Infection status	Experiment
1	Apo1	**Apo**	**#1**
2	Apo2	**Apo**	**#1**
3	Apo3	**Apo**	**#1**
4	Apo4	**Apo**	**#2**
5	Apo5	**Apo**	**#2**
6	Apo6	**Apo**	**#2**
7	Lux1	**Lux**	**#1**
8	Lux2	**Lux**	**#1**
9	Lux3	**Lux**	**#1**
10	Lux4	**Lux**	**#2**
11	Lux5	**Lux**	**#2**
12	Lux6	**Lux**	**#2**
13	WT1	**WT**	**#1**
14	WT2	**WT**	**#1**
15	WT3	**WT**	**#1**
16	WT4	**WT**	**#2**
17	WT5	**WT**	**#2**
18	WT6	**WT**	**#2**

A Between-library comparison

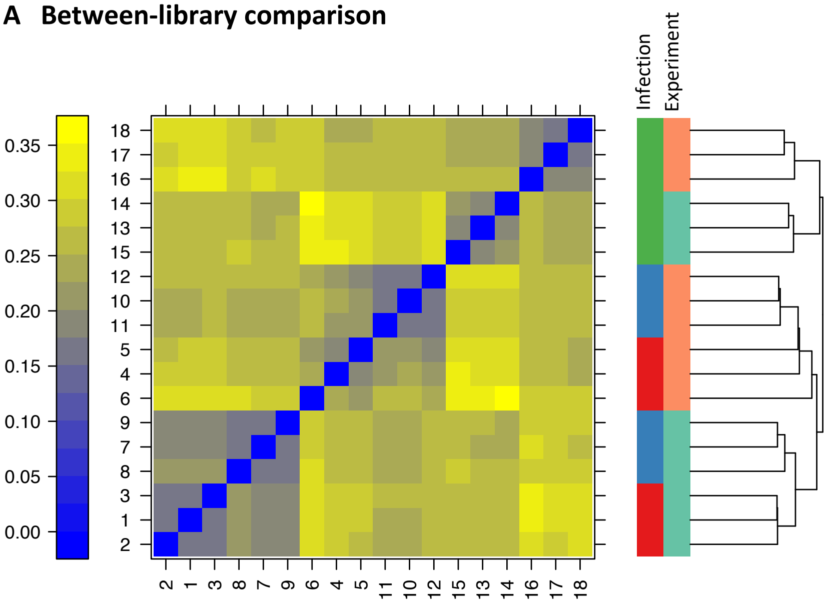

B Variance mean dependence

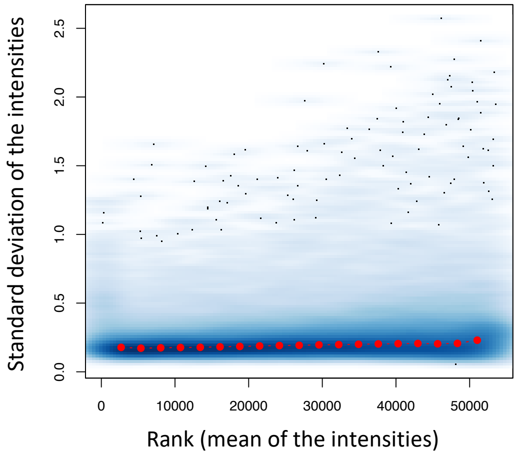

C Library intensity distributions

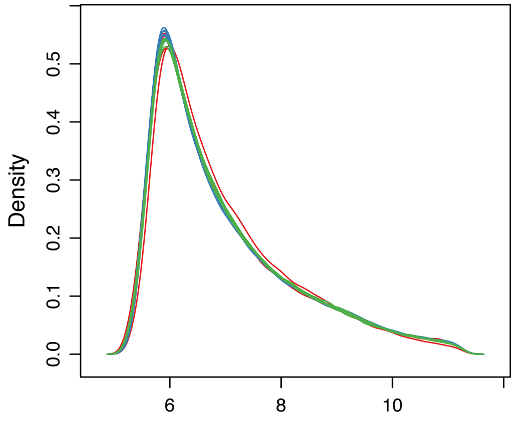

D Individual library quality

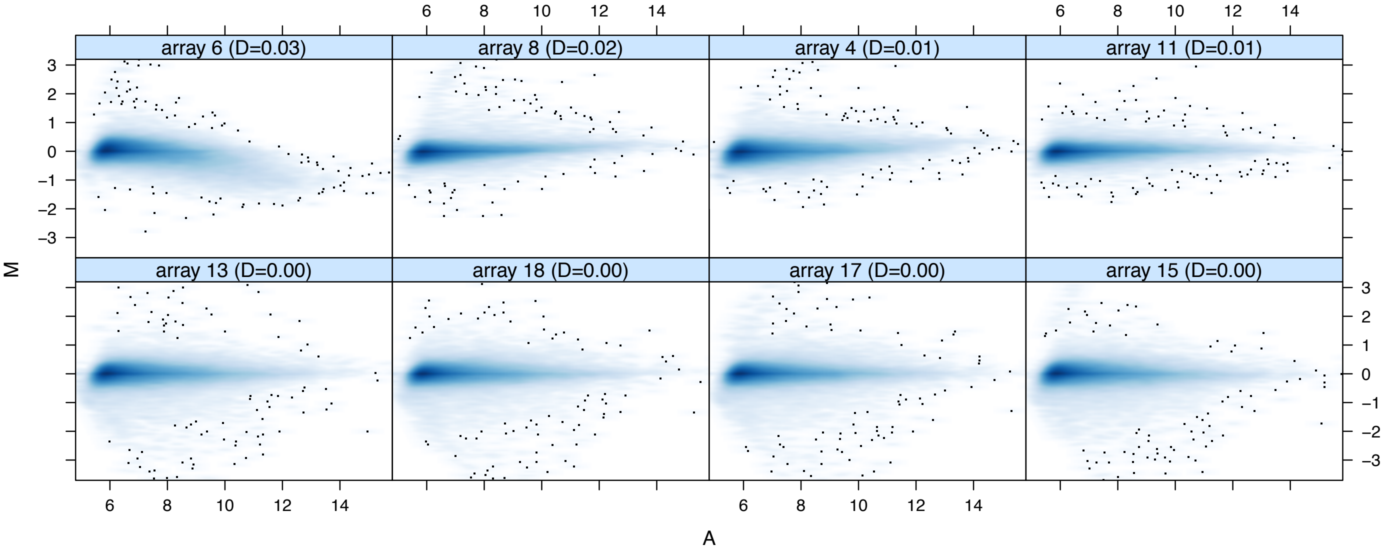

E Validation of normalized Illumina counts by quantitative RT-PCR

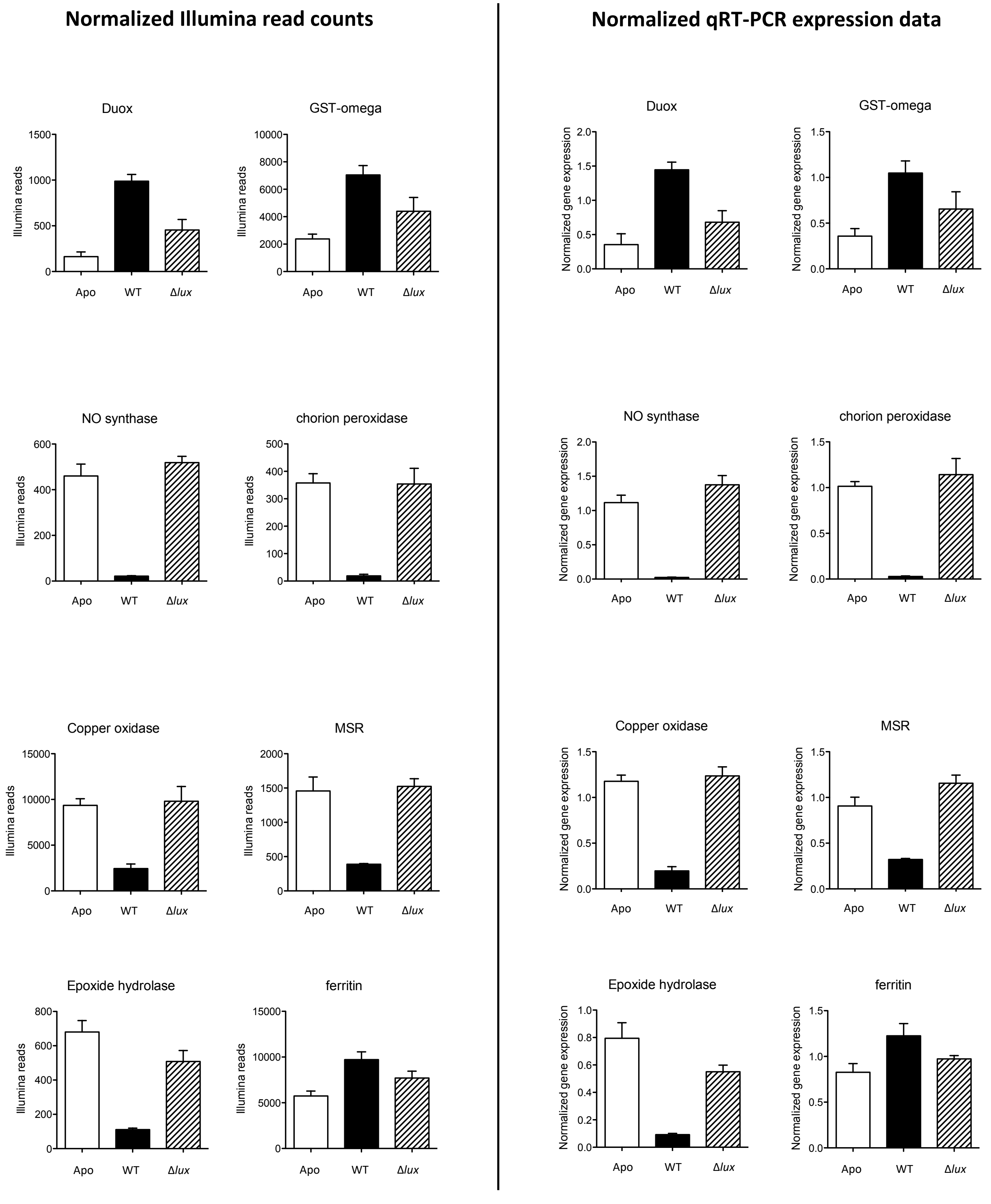

Supplement: FIG S1 [file sys005182266sf1.pdf]
