# Supplementary material for: Persistent Interactions with Bacterial Symbionts Direct Mature-Host Cell Morphology and Gene Expression in the Squid-Vibrio Symbiosis
Source: mSystems. 2018 Oct 2;3(5):e00165-18. doi: 10.1128/mSystems.00165-18 (PMC6172772; doi:10.1128/mSystems.00165-18)

Supplementary File 2

A Size distribution of assembled isogroups before size/coverage filtering process

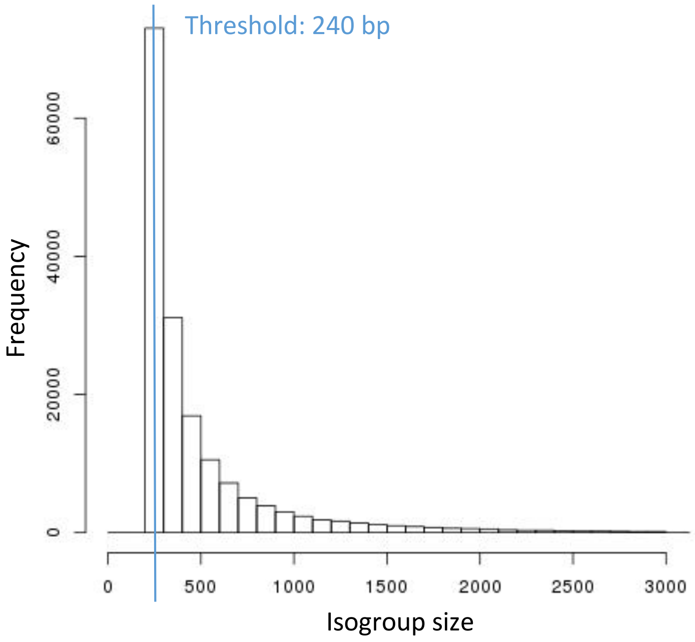

B Read coverage distribution of assembled isogroups before size/coverage filtering process

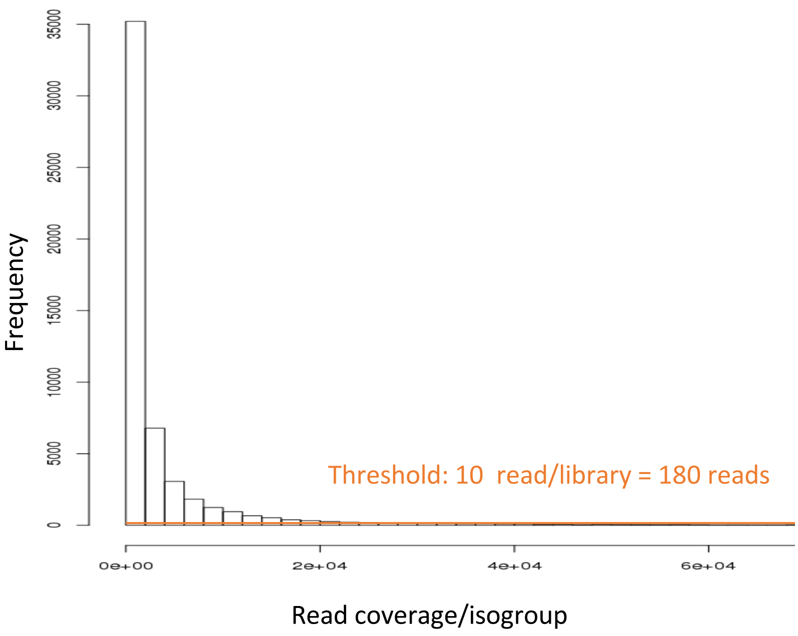

C Comparison between two BLAST procedures

Number of isogroups with BLAST results
(22 194/53719 = 41.3% of total transcripts)

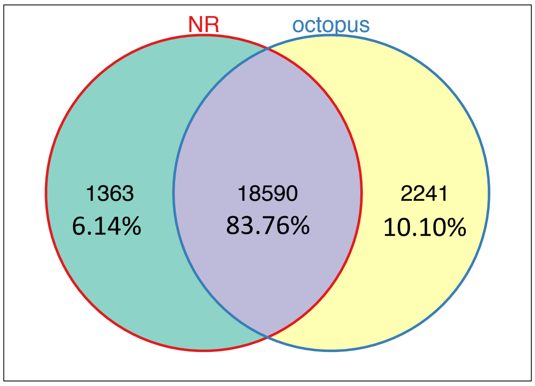

NR = BLAST result against nr
octopus = BLAST result against the *Octopus bimaculoides* transcriptome

Number of isogroups with GO annotation
(13 942 = 62.8% of BLASTed transcripts)

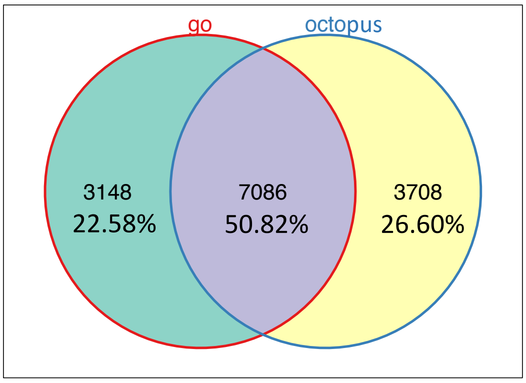

go = GO annotation *via* Blast2go from nr BLAST
octopus = GO annotation from the *Octopus bimaculoides* annotation

Number of isogroups with PFAM annotation
(16357 = 73.7% of BLASTed transcripts)

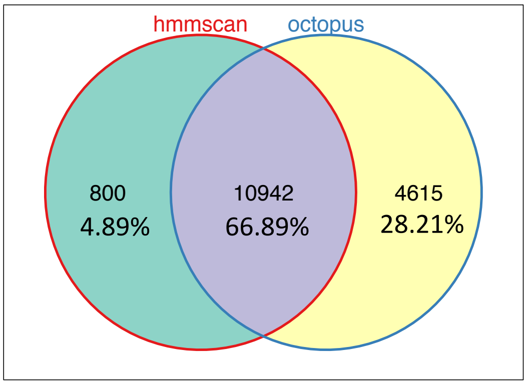

hmmscan = prediction of PFAM motifs on predicted ORFs
octopus = PFAM terms from the *Octopus bimaculoides* annotation

D Sequence similarity and e-value distribution from the BLASTs against nr and against the *Octopus* transcriptome

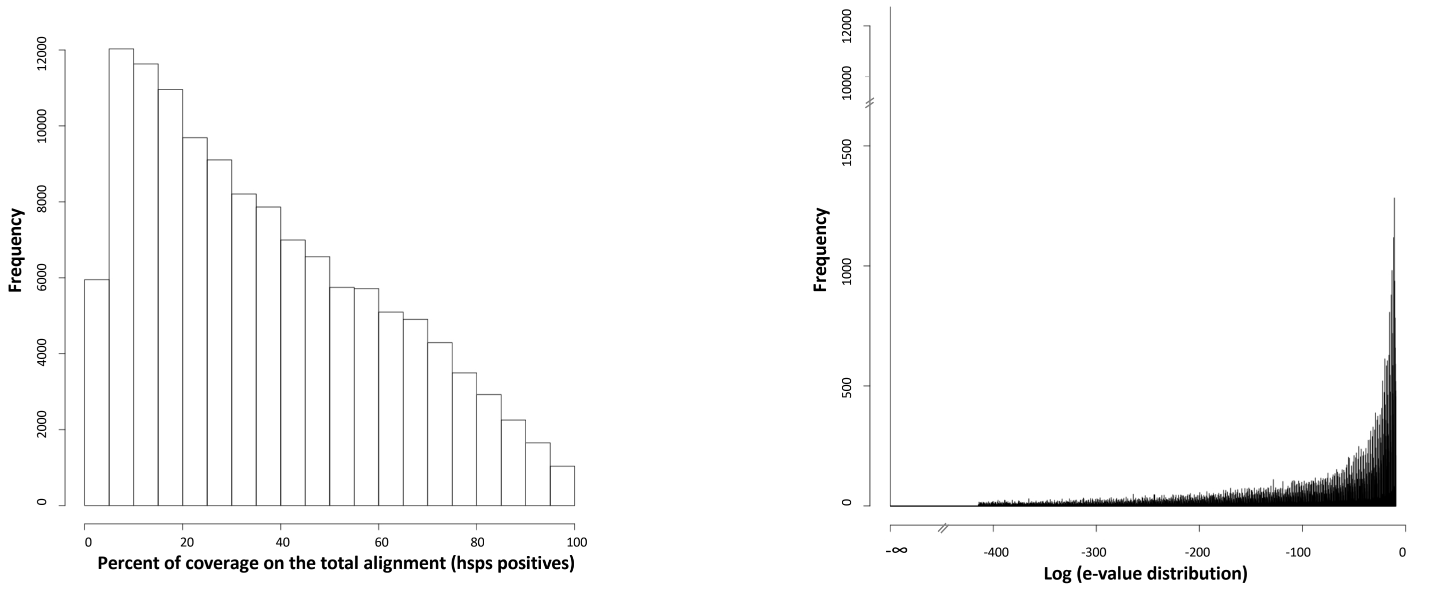

E Species distribution from the BLAST against nr

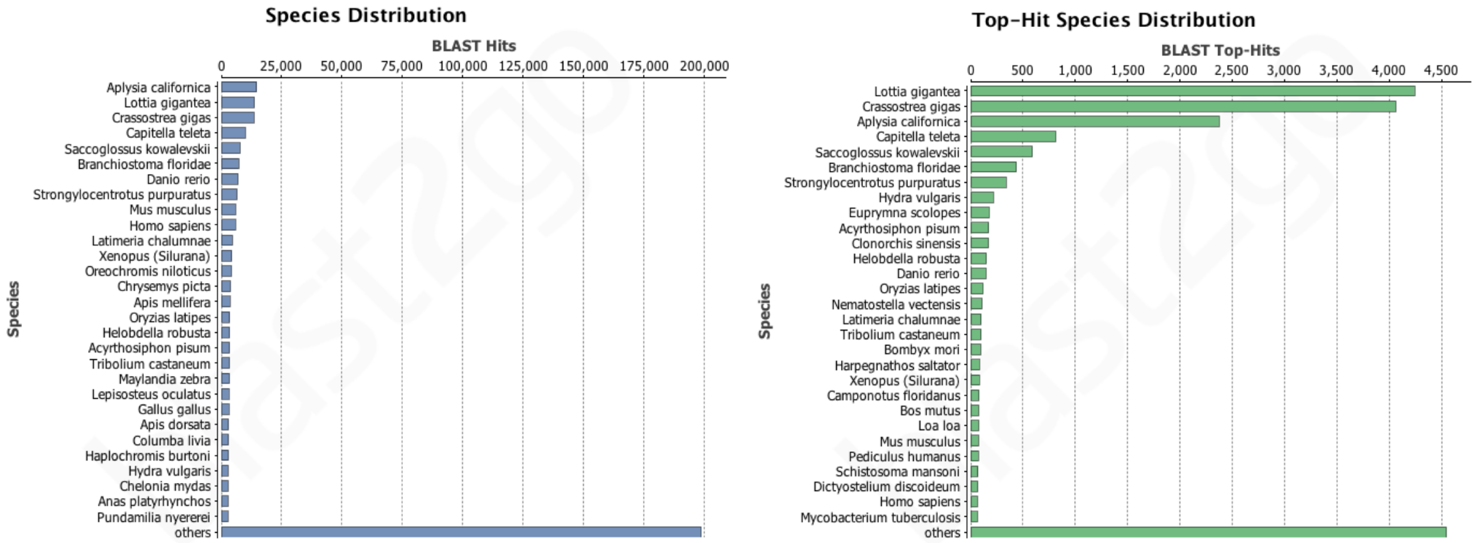

Supplement: FIG S2 [file sys005182266sf2.pdf]
